# Enhanced Permutation Tests via Multiple Pruning

**DOI:** 10.3389/fgene.2020.00509

**Published:** 2020-06-25

**Authors:** Sangseob Leem, Iksoo Huh, Taesung Park

**Affiliations:** ^1^Department of Statistics, Seoul National University, Seoul, South Korea; ^2^College of Nursing and Research Institute of Nursing Science, Seoul National University, Seoul, South Korea

**Keywords:** permutation test, multiple hypothesis testing, pruning, big multi-omics data, GWAS

## Abstract

Big multi-omics data in bioinformatics often consists of a huge number of features and relatively small numbers of samples. In addition, features from multi-omics data have their own specific characteristics depending on whether they are from genomics, proteomics, metabolomics, etc. Due to these distinct characteristics, standard statistical analyses using parametric-based assumptions may sometimes fail to provide exact asymptotic results. To resolve this issue, permutation tests can be a way to exactly analyze multi-omics data because they are distribution-free and flexible to use. In permutation tests, *p*-values are evaluated by estimating the locations of test statistics in an empirical null distribution generated by random shuffling. However, the permutation approach can be infeasible when the number of features increases, because more stringent control of type I error is needed for multiple hypothesis testing, and consequently, much larger numbers of permutations are required to reach significance. To address this problem, we propose a well-organized strategy, “ENhanced Permutation tests via multiple Pruning (ENPP).” ENPP prunes the features in every permutation round if they are determined to be non-significant. In other words, if the feature statistics from the permuted datasets exceed the feature statistics from the original dataset, beyond a predetermined threshold, the feature is determined to be non-significant. If so, ENPP removes the feature and iterates the process without the feature in the next permutation round. Our simulation study showed that the ENPP method could remove about 50% of the features at the first permutation round, and, by the 100th permutation round, 98% of the features had been removed and only 7.4% of the computation time with the original unpruned permutation approach had elapsed. In addition, we applied this approach to a real data set (Korea Association REsource: KARE) of 327,872 SNPs to find association with a non-normally distributed phenotype (fasting plasma glucose), interpreted the results, and discussed the feasibility and advantages of the approach.

## Introduction

Unlike typical big data, big data in bioinformatics consists of huge numbers of features and relatively small numbers of samples. For example, the data from genome-wide association studies (GWAS) contain at least thousands of samples and several hundred thousands of single nucleotide polymorphisms (SNPs) (Manolio, 2010). In the case of transcriptomic analysis for finding differently expressed genes, tens of thousands of genes are tested from only hundreds of samples at most (McLachlan et al., 2005). In epigenomic data, such as DNA methylation, the number of features (e.g., CpG sites) varies from tens of thousands to several million according to profiling techniques and their resolution (Bibikova et al., 2011; Adusumalli et al., 2014). Moreover, not only large numbers of features but also various characteristics of the features are important points to be considered. For example, in genomic data, such as SNPs, a feature is represented as a count of a minor allele at a genomic locus in each individual. In transcriptome data sets, gene expression levels are represented as continuous and positive real values measured from microarray spot intensities. In the case of epigenomics data, the DNA methylation levels of loci can be provided as a ratio between read counts of C and read counts of C and T. In addition, proteomics and metabolomics data provide marker intensities from mass-spectrometry-based approaches. Therefore, detecting association between phenotypes and biomarkers using standard statistical approaches may sometimes be inaccurate, as many of these are based on parametric assumptions that require specific properties of the features. Although several remedies have been proposed in terms of parametric approaches (Thygesen and Zwinderman, 2004; Lin et al., 2008; Park and Wu, 2016), they are naturally asymptotic ones and still possibly have type 1 error inflation or low power.

As an alternative to these issues, the permutation test (Pitman, 1937; Annis, 2005) has become a popular approach for analyzing multi-omics data because it can be used regardless of the shape of distribution of the biomarkers’ expression and uses a simple algorithm. In the permutation test, a *p*-value is assessed through evaluating the relative rank of the observed test statistic in an empirical null distribution of the test statistic generated by random shuffling. The permutation test has already been used in some omics analysis. For example, in GWAS, the permutation test is used for adjusting for multiple tests (Browning, 2008), considering biological structures (Pahl and Schäfer, 2010), and identifying gene-gene interactions (Ritchie et al., 2001; Greene et al., 2010). In next-generation sequencing data analysis, rare variants have been identified by permutation test for association with a phenotype (Madsen and Browning, 2009) and as a significance test of structural models (Lee et al., 2016; Kim et al., 2018). In integration analysis of multi-omics data, the permutation test is used for finding edges in the integrated network (Jeong et al., 2015) and significance testing of an aggregated unit with a structure (Kim et al., 2018). In metagenome studies, the permutation test is used for testing differences between distances of groups (Chen et al., 2012), finding differentially abundant operational taxonomic units (Anderson, 2005), and finding differentially abundant genomic features (Paulson et al., 2011).

However, a major obstacle to the permutation test is its large computation time, because the smallest *p*-value that a permutation test can reach is inversely proportional to the permutation time. Therefore, if a data set has a large number of features, it requires a large number of permutations to detect significantly associated features because larger numbers of features require more stringent type 1 error control in terms of multiple hypothesis testing correction. For example, if a researcher wants to test an association between 5.0 × 10^5^ SNPs and a specific phenotype, the *p*-value threshold will be 1.0 × 10^–7^ [0.05/(5.0 × 10^5^) by Bonferroni correction]. To achieve such a stringent *p*-value threshold, the number of permutations must be at least 1.0 × 10^7^−1 for each SNP, and the total computation time for all features is impractical. Considering that only significant features are of general interest to researchers, pruning insignificant features can be a way to resolve the issue.

Therefore, in this study, we propose a well-organized strategy, ENhanced Permutation tests via multiple Pruning (ENPP). The key idea of ENPP is simple. When the number of features is large, the *p*-value threshold is very low due to multiple testing correction. In most cases, if a feature is reported to be significant, its observed test statistic value should be more extreme than those from permuted data sets. On the other hand, if a feature has more than a set number of instances of having larger statistics from permuted data sets, it can be regarded as a feature with significantly less chance of being significant, and ENPP prunes the feature during a certain permutation round. In other words, ENPP specifically removes non-significant features and continues the permutation procedures with the remaining features, which can then be candidates for a predetermined significance level. This approach can reduce total permutation time to a feasible level compared to ordinary permutation approaches that conduct the same number of permutation tests on all features. Herein, we show that ENPP can remove about 50% of features in the first permutation round and requires, at the 100th permutation round, only 7.4% of the computation time needed for the unpruned permutation approach. This relative proportion of computation time becomes smaller as the iteration time increases. In addition, we applied our approach to a real data set (Korea Association REsource: KARE) (Cho et al., 2009) containing 327,872 SNP features and a non-normally distributed phenotype (fasting plasma glucose, FPG) for validation of our approach in terms of feasibility and usefulness.

## Materials and Methods

### Data Set

For real data analysis, we chose a Korean GWAS data set collected since 2007 by The Korean Association REsource (KARE) project (Cho et al., 2009). In this project, all participants were recruited from either of two region-based cohorts (rural Ansung and urban Ansan). The total number of participants was 10,038 (5,018 from Ansung and 5,020 from Ansan), and they were all genotyped, using genomic DNA from peripheral blood, using the Affymetrix (Santa Clara, CA, United States) Genome-Wide Human SNP array 5.0, containing 500,568 SNPs. For quality control, we followed the same process used in a previous study (Oh et al., 2016). As a result, we finally obtained 8,842 individuals and 327,872 SNPs, and the processed data set was used in our real data analysis. The study was reviewed and approved by the Institutional Review Board of Seoul National University (IRB No. E1908/001-004).

### ENPP Approach

Suppose that there are *N* samples, each with a dependent variable *Y*, and *J* features *X*_1_,…,*X*_*J*_, representing features from a multi-omics data set. In general, for a significance test of association between a specific *X*_*j*_ and *Y*, the null distribution of the test statistic *S* consists of test statistics from permuted data sets, and we call the statistics *s*_*r*_, where *r* = 1,2,…,*R*, with *R* denoting the total number of permutation rounds for the feature. Then, the observed value, *s*_*obs*_ (i.e., the original value of the test statistic, *S*) is compared to the null distribution of *S*, and the significance is assessed by the proportion of *s*_*r*_ values more extreme than *s*_*obs*_. For exact generation of the null distribution, *N*! iterations are required. However, when *N*! is too large, *R* iterations of random shuffling (*R*) (*R*≪*N*!) are generally used for assessing computational feasibility in terms of Monte-Carlo estimation. A finding that a *s*_*obs*_ value is larger than the simulated *s*_*r*_ values implies that the test is more supportive of the alternative hypothesis, and the *p*-value is then calculated by the following equation:

(1) $$P_{p\,e\,r\,m}=\frac{1+\sum_{r=1}^{r=R}I\left(s_{o\,b\,s}\leq s_{r}\right)}{R+1},$$

where I(⋅) is an indicator function, and +1 in the numerator and denominator can be omitted.

When the number of features is multiple, the *p*-value threshold should be adjusted for a multiple testing comparison. For example, a typical *p*-value threshold is 0.05, and, if there are 1,000 features for association tests, then the *p*-value threshold becomes 0.05/1,000, for the Bonferroni correction. In other words, when a feature has a *p*-value smaller than this adjusted *p*-value threshold it is reported as significant. Therefore, the possibility of I(⋅) = 1 (more extreme than the observed statistic value) is extremely low for this feature. On the other hand, if I(⋅) = 1 frequently appears in a feature, the *p*-value of the feature may be closer to 1, meaning that it may not be significant and would therefore be of no interest to researchers. Let *p*_*raw*_ be an unadjusted *p*-value threshold (e.g., 0.05) and *p*_*adj*_ be an adjusted *p*-value threshold, for each feature, after the multiple testing correction (e.g., 0.05/J by Bonferroni correction). *p*_*adj*_ is then the significance level for which we need to detect significant features, and the decision of whether or not to prune a feature, in any specific round, is based on the hypothesis that:

(2) $$\text{H}\,0:p=p_{a\,d\,j},\text{and}\,\mathrm{H1}:p>p_{a\,d\,j},$$

where p implies the true p-value from the permutation approach. In the hypothesis, the significance level for the test needs to be determined, and we call the threshold p_*prun*_. For the hypothesis test, a binomial test can be used, and, based on p_*adj*_ and p_*prun*_, we can set an integer C_*prun*_ that satisfies p_*prun*_ in a permutation round. Therefore, C_*prun*_ is a variable that depends on permutation numbers, while p_*adj*_ and p_*prun*_ are fixed values for the whole pruning process. Consequently, using this rule, EPNN counts in how many cases a feature has a more extreme test statistic than its observed test statistic value in each permutation round. If a feature is equal to or greater than C_*prun*_ in a round, it is removed from the next permutation round. The following is a detailed explanation of the parameter determination.

Let us assume that p_*adj*_ = 5 × 10^–5^, which is equivalent to a threshold Bonferroni correction with 1,000 features, and p_*prun*_ = p_*adj*_. In addition, if we let *p*_*k—r*_ denote a probability of observing at least a number *k* of test statistics values more extreme than the observed test statistics at the *r*th permutation round, then $p_{k|r}=\sum_{t=k}^{t=r}\left(\begin{array}{c} r\\ t \end{array}\right)\,p_{a\,d\,j}^{t}\,{\left(1-p_{a\,d\,j}\right)}^{r-t}$. Therefore, if the p-value of a feature is significant, then *p*_*k—r*_ should be equal to or smaller than p_*prun*_. As an illustration, consider the first permutation round. Based on a setting of p_*adj*_ = 5 × 10^–5^, two probabilities, *p*_0|1_,*p*_1|1_, are given. Because we set *p*_*prun* =_*p*_*adj*_,*p*_0|1_ will be 1 and *p*_*1—1*_ will be *p*_*adj*_, implying that *C*_*prun*_ = 1 is in the first round. For the second round, there are three probabilities, *p*_0|2_,*p*_1|2_ and *p*_2|2_, that can be easily computed. In this case, *p*_1|2_ = 1×10^−4^ > *p*_*prun*_*p*_2|2_ = 10^−9^ < *p*_*prun*_. Therefore *C*_*prun*_ will be 2 for the second round. In this manner, we can obtain *C*_*prun*_ for all permutation rounds conducted. We will show the properties of the parameters in the next section.

## Results

### Simulation Analysis

In this section, we evaluated the advantages of ENPP compared to a strict permutation approach, including its need for only very few counts for rejecting and removing non-significant features. As a consequence of this attribute, ENPP can greatly reduce total computation time to a feasible level compared to an unpruned permutation approach. To show the desired properties, we artificially generated data sets whose features did not associate with a feature. When the Bonferroni threshold was applied and *p*_*raw*_ = 0.05, the first example had $p_{a\,d\,j}^{1}$ = 0.05/1,000 and the second example had $p_{a\,d\,j}^{2}$ = 0.05/(5 × 10^5^). In addition, we also assumed that *p*_*prun*_ = *p*_*adj*_ for both examples.

### Distribution of *C_prun_*

**FIGURE 1:**
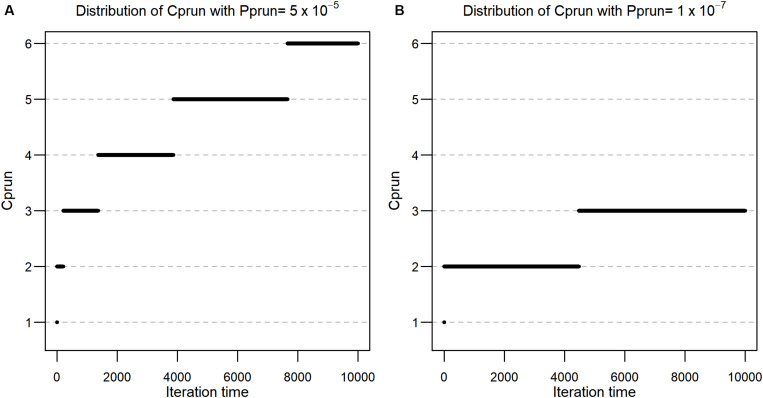
**(A)** Distribution of *C*_*prun*_ with *P*_*prun*_ = 5 × 10^–5^. **(B)** Distribution of *C*_*prun*_ with *P*_*prun*_ = 1 × 10^–7^.

Firstly, we investigated the distribution of *C_prun_* values according to each permutation round for $p_{a\,d\,j}^{1}$, and $p_{a\,d\,j}^{2}$, respectively. Using the formula described in the methods, *C_prun_* values were calculated for *r* = 1,2,…, 10,000, and the resulting values are shown in Figure 1A, which also shows that the values of *C_prun_* for $p_{a\,d\,j}^{1}$ are at most 6 in the 10,000th round. This implies that the threshold is not hard to satisfy and that we can reduce a large proportion of the number of features at each permutation round. In the case of $p_{a\,d\,j}^{2}$, *C_prun_* becomes smaller (Figure 1B). In detail, *C_prun_* is 1 for *i* = 1, 2 for i ∈ [2, 4,473], and 3 for i ∈ [4,474, 10,000], implying that smaller *p*_*adj*_ values provide smaller *C_prun_* values, although *p*_*prun*_ is proportional to *p*_*adj*_.

### Pruning Rates and Computational Efficiency in Each Permutation Round

Based on the *C_prun_* values calculated above, we also evaluated the pruned proportion of the total features for each permutation round. Suppose that the p-value of a feature has a uniform distribution, meaning that the feature has no association with a phenotype. In this setting, the pruned proportion of features depends only on *C_prun_*. For example, at the first round, for *C*_*prun*_(1) = 1, the proportion of pruned features will be $\int_{0}^{1}p\,dp=\frac{1}{2}$. At the second round, for *C*_*prun*_(2) = 2, no pruning will happen, because the event that *C*_*prun*_(1) = 1 includes the event that *C*_*prun*_(2) = 2. At the third round of permutation, for *C*_*prun*_(3) = 2, the expected pruning proportion after the permutation will be:

$$\int_{0}^{1}\left(1-p\right)\,p^{2}\,dp=\int_{0}^{1}\left(p^{2}-p^{3}\right)\,dp=\frac{1}{3}-\frac{1}{4}=\frac{1}{12}.$$

**FIGURE 2:**
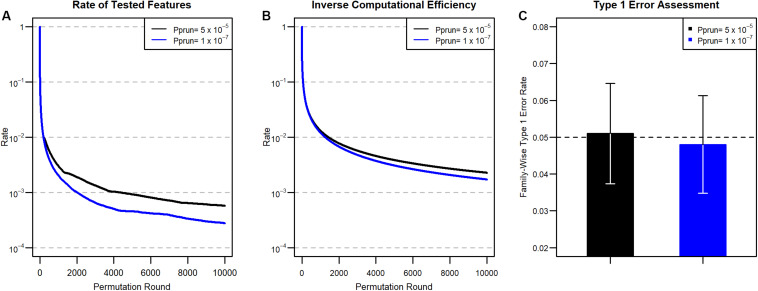
**(A)** Proportion of tested features at each round after pruning for the two *P*_*prun*_ values in Figure 1. **(B)** Inverse of computational efficiency (ICE) for 2A. **(C)** Type 1 error results. We divided the number of false-positive features by 1,000 to obtain the family-wise type 1 error rate. 95% confidence intervals of the estimated type 1 errors are also provided.

In other words, at the first permutation, $\frac{1}{2}$ of the features are expected to be pruned, and $\frac{1}{12}$ of the features are additionally pruned after the third round. In this manner, the expected proportions of remaining features after pruning from 1 to 10,000 permutation rounds are calculated using the *C_prun_* values (Figure 1), and the results are described in Figure 2. Because the cumulative pruning proportion is not easily derived by numerical calculation, we estimated the proportion by simulation using variables from a Bernoulli distribution, with the probability for success taken from a uniform distribution U(0,1). In Figure 2A, only about 2% of features remain after the 100th permutation round in both *p*_*prun*_ settings, thus greatly reducing the number of tests for the data set at the round. However, as *C_prun_* becomes different, the remaining proportions also become different. For example, at the 1000th permutation round, 0.3% of total features remained for $p_{a\,d\,j}^{1}$ and 0.2% for $p_{a\,d\,j}^{2}$. The ratio between the two proportions became larger at the 10,000th permutation round, with 0.057% for the former, $p_{a\,d\,j}^{1}$, and 0.028% for the latter, $p_{a\,d\,j}^{2}$. These results reflect the differences of *C_prun_* provided in Figure 1.

We next assessed computational efficiency by comparing the total permutation time for ENPP to that for the original, unpruned permutation test. The efficiency is represented as a ratio between the number of tests in the original unpruned permutation approach and the cumulative number of tests in the ENPP approach. The total permutation time for a given permutation round in ENPP is calculated by accumulating all permutation times of earlier permutation rounds. Therefore, larger computational efficiencies imply a large timesaving advantage for ENPP analysis. For example, during the first round, there is no reduction of permutation time, but for the second and third permutation rounds, ENPP needs only $\frac{1}{2}$ the computations compared to the original unpruned permutation tests, and $\frac{5}{12}$ the permutations are needed for the fourth round. Therefore, computational efficiency will be $\frac{1}{1}=1$ for the first permutation round, and $\frac{1+1}{1+\frac{1}{2}}=\frac{4}{3},\frac{1+1+1}{1+\frac{1}{2}+\frac{1}{2}}=\frac{3}{2},\frac{1+1+1+1}{1+\frac{1}{2}+\frac{1}{2}+\frac{5}{12}}=\frac{48}{29}.$ for the second, third, and fourth permutation rounds, respectively. The Inverse Computational Efficiency (ICE) for each permutation round is summarized in Figure 2B. In Figure 2B, ICE does not seem to decrease as fast as the remaining proportion, as shown in Figure 2A, due to the fact that permutation times of precedent rounds accumulate in estimating computational efficiency. Compared to the ordinary unpruned permutation test, only about 7.4% of the computation time is needed at the 100th permutation round in both settings, because they have the same numbers for *C_prun_* and the same resulting remaining proportions. However, as in the remaining proportion of features, ICE became more different in terms of ratios between the two settings as the permutation round progresses. For example, at the 1000th permutation round, ICE is 1.3% for *p*_*prun*_ = 5 × 10^–5^ and 1.2% for *p*_*prun*_ = 1 × 10^–7^. However, in the 10,000 iteration, 0.23% is needed for the former, *p*_*prun*_ while 0.17% is needed for the latter, *p*_*prun*_. Thus, the overall computational efficiency improves as the iteration round progresses because the remaining rate of the features grows smaller, and smaller *p*_*prun*_ requires less computation.

On the other hand, we assessed the type 1 error rate of non-associated features from the ENPP approach. For $p_{a\,d\,j}^{1}$ and $p_{a\,d\,j}^{2}$, we generated 10^6^ and 5 × 10^8^ non-associated features from the Bernoulli distribution so that the expected numbers of features with type 1 error are 50 in both settings. We first applied the pruning process to the non-associated features and then the full permutation approach to the remaining unpruned features. After the full permutation approach had been applied, we counted how many non-associated features were found significant at the given significance levels. The type 1 error rates are summarized in Figure 2C, showing that the ENPP approach controls the type 1 error well.

### Real Data Analysis

**FIGURE 3:**
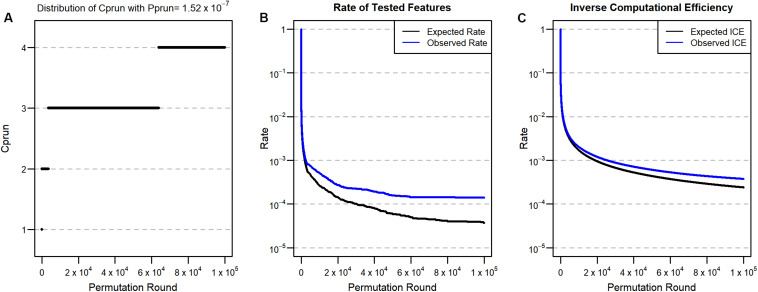
**(A)** Distribution of *C*_*prun*_ for the real data set with *P*_*prun*_ = 1.52 × 10^–7^. **(B)** Proportion of tested features after pruning. **(C)** ICE for *P*_*prun*_ = 1.52 × 10^–7^. Black lines are expected values from the simulation, and blue lines are observed values from the real data analysis.

We next applied our approach to a real genome-wide data set (Korea Association REsource: KARE), which has 327,872 SNPs from each of 8,842 individuals (Cho et al., 2009). In order to detect significant SNP features at the Bonferroni significance level in the data set, the ordinary permutation approach (without ENPP) requires at least (1/0.05) × 327,872^2^ = 2.15 × 10^12^, a computationally impractical number of tests. Therefore, using a pruning approach for this data set becomes inevitable when the permutation approach is used. For the application of ENPP, we set *p*_*raw*_ = 0.05 and *p*_*prun*_ = *p*_*adj*_ = 0.05/327,872 = 1.52 × 10^–7^, and the corresponding *C_prun_* is calculated and described in Figure 3A. Here, we set the number of iterations to 100,000 because simulation analysis found that the remaining proportion of features was 3.7 × 10^–5^ at the 100,000th round and the corresponding expected count of remaining features was 3.7 × 10^–5^ × 327,872 = 12.13 if all features were assumed not to associate with a phenotype. We selected fasting plasma glucose (FPG) as a phenotype because its distribution is very highly skewed (skewness = 5.32) and the skewness is still high (=2.71) (Kim, 2013) even after log-transformation. Consequently, we expected that this property may produce results that differ between a parametric approach and a permutation approach. For the association analysis, we used age, gender, and living regions as covariates, and we assumed that the genotype of the SNP features has an additive effect on the phenotype. As a test statistic for the permutation test, we used a t-statistic for the genotype effect.

Based on the expected remaining proportion of the features, we found ICE to be 2.4 × 10^–4^ at the 100,000th permutation round (Figure 3C), meaning that we needed only 24 times more computation compared to the parametric linear regression approach. This number of permutation tests can be done in a few days, even in a single thread. After implementing the 100,000th iteration of ENPP with the real data set, we plotted the number of remaining features (Figure 3B) and the ICE (Figure 3C) in each round. Those results showed that 46 SNP features remained and that the computational efficiency was 3.7 × 10^–4^, implying that some SNP features were candidates for significant features. For each of 46 SNP features, we implemented a 3×10^7^−1 permutation test to provide a *p*-value not only for Bonferroni correction but also for a genome-wide significance of 5 × 10^–8^ (Xu et al., 2014). After implementation of the test, we found that five SNP features passed the Bonferroni threshold, and two SNPs also passed for genome-wide significance (Table 1). On the other hand, the parametric approach found four SNPs for Bonferroni correction, and two SNPs passed genome-wide significance. However, only three SNPs overlapped for the former threshold, and one SNP overlapped for the latter one. To determine substantial differences of *p*-values between the two approaches, we used an exact binomial test (Clopper and Pearson, 1934) that regarded *p*-values from the parametric approach as a null hypothesis *p*-value for the permutation results. From the test, we found that only one SNP (rs7197218G in chromosome 16) showed a significant difference between the two results (Table 1). This SNP showed a more conservative result from the permutation approach; this result may come from type 1 error inflation in the parametric test in the presence of very low minor allele frequency and large differences of variance between FPG values with and without the minor allele (Zimmerman, 2004).

**TABLE 1:** 6 SNPs selected from either parametric (linear regression) or non-parametric (ENPP) tests at a Bonferroni significance level *p* = 1.52 × 10^–7^.

CHR	SNP id	MAF	*P*-value from linear regression	*P*-value from permutation	*P*-value from comparison between the two values
6	rs9348440T	0.478	1.63 × 10^–7^	1.33 × 10^–7^	1
6	rs6456368C	0.480	1.54 × 10^–7^	1.00 × 10^–7^	0.640
6	rs10946398C	0.479	8.35 × 10^–8^	6.67 × 10^–8^	1
6	rs7754840C	0.479	4.93 × 10^–8^	3.33 × 10^–8^	1
6	rs9460546G	0.481	5.45 × 10^–8^	3.33 × 10^–8^	1
16	rs7197218G	0.014	4.81 × 10^–8^	7.33 × 10^–7^	<2.2 × 10^–16^

*Here, we provide information for SNP features such as chromosome, SNP id, and minor allele frequency (MAF) and the *p*-values from both tests. In the last column of the table, we also include the results of an exact binomial test for permutation results based on the null hypothesis that the *p*-value of the permutation test is the same as the results from the parametric approach.*

## Discussion

For the analysis of multi-omics data, the permutation test has been popularly used because it is non-parametric and flexible to use. However, the main drawback of this approach is that it may require such a large number of tests as to make it infeasible, especially for data sets with large numbers of features and a Bonferroni-corrected significance level. To resolve this issue, we proposed a well-organized strategy, ENhanced Permutation tests via multiple Pruning (ENPP), for enhanced permutation tests, using the idea of pruning. ENPP investigates the features at every permutation round and removes them if they have less chance of being significant. Our empirical study showed that the ENPP method could remove about 50% of the number of features at the first permutation round and required only 7.4% of the total computation time at the 100th permutation round as is needed by an unpruned approach. Moreover, in real data analysis, on a data set of 327,872 SNP features, our approach was found to greatly reduce computational burden to a feasible level, and the analysis results seemed more reliable than the results from a parametric approach because they were not affected by a specific assumption of a null distribution. Interestingly, we found that the number of tests conducted in the ENPP process was much smaller than the number in the final evaluation of the 46 SNP features to obtain precise *p*-values. In the pruning process of real GWAS data, about 1.2 × 10^7^ permutations were needed, while in parallel, the full permutation analysis required about 1.4 × 10^9^ iterations. Since the pruning process and the full permutation process are performed on each feature independently, they can easily be parallelized. We believe that parallelism has a large impact on the full permutation process because the full permutation process seems to take much more computing time than the pruning process in our real data analysis. Therefore, with the help of parallel computing, our ENPP approach can easily handle, without computational burden, larger data sets such as human methylation data with 2 × 10^7^ CpG site features.

Our EPNN algorithm is also flexible for pruning processes. Researchers can modify *p*_*adj*_ and *p*_*prun*_ as they want. In this study, we set *p*_*adj*_ = *p*_*prun*_, with *p*_*adj*_ from a Bonferroni correction, and conducted 100,000 ENPP permutations. These settings could be interpreted with the number of expected significant features and the number of tests of the features, considering that summation of the actual significance level, calculated for *C*_*prun*_, from the first round to the 100,000th round is 2.66 × 10^–3^, and it admits 0.05/(2.66×10^–3^) ≈ 18 truly significant features at the Bonferroni threshold. In other words, if there are 18 or fewer significant features, at p = 1.52×10^–7^, we can control the probability of falsely pruning any significant features under 0.05. This assumption of the number of the significant features is reasonable, considering that only a few features may satisfy Bonferroni cutoff in general and that our analysis results in both parametric and permutation approaches found only four or five SNPs, respectively. In addition, researchers may sometimes be interested not only in features for a specific Bonferroni significance level but also in a *p*-value distribution of whole features. For this purpose, ENPP can be applied after some number of unpruned permutation rounds, such as 100, so that more precise *p*-values can be obtained, even for non-significant features, and the results can be used in false discovery rate (FDR) approaches (Benjamini and Hochberg, 1995) or in combining *p*-value approaches for some group-wise testing such as gene- or pathway-wise significance tests (Subramanian et al., 2005). Our ENPP approach will help many researchers achieve precise *p*-values in a feasible time, even for datasets with a large number of features. A brief R script for performing ENPP is provided for SNPs at http://statgen.snu.ac.kr/software/ENPP. This will enable more accurate decisions based on the statistical results.

## Data Availability Statement

The data will be publicly distributed by the Distribution Desk of the Korea Biobank Network (https://koreabiobank.re.kr/), to whom data requests should be directly made. Any inquiries should be sent to admin@koreabiobank.re.kr.

## Ethics Statement

The study was reviewed and approved by the Institutional Review Board of Seoul National University (IRB No. E1908/001-004). The patients/participants provided their written informed consent to participate in this study.

## Author Contributions

SL, IH, and TP developed the algorithm. SL conducted the simulation study and wrote the manuscript. IH conducted real data analysis and wrote the manuscript. TP supervised the whole research project. All authors contributed to the article and approved the submitted version.

## Conflict of Interest

The authors declare that the research was conducted in the absence of any commercial or financial relationships that could be construed as a potential conflict of interest.
